# Supplementary material for: C57bl/6 Mice Show Equivalent Taste Preferences toward Ruminant and Industrial Trans Fatty Acids
Source: Nutrients. 2023 Jan 24;15(3):610. doi: 10.3390/nu15030610 (PMC9918975; doi:10.3390/nu15030610)
Supplement: Supplementary file 1 [file nutrients-15-00610-s001.zip › nutrients-2135283-supplementary.pdf]

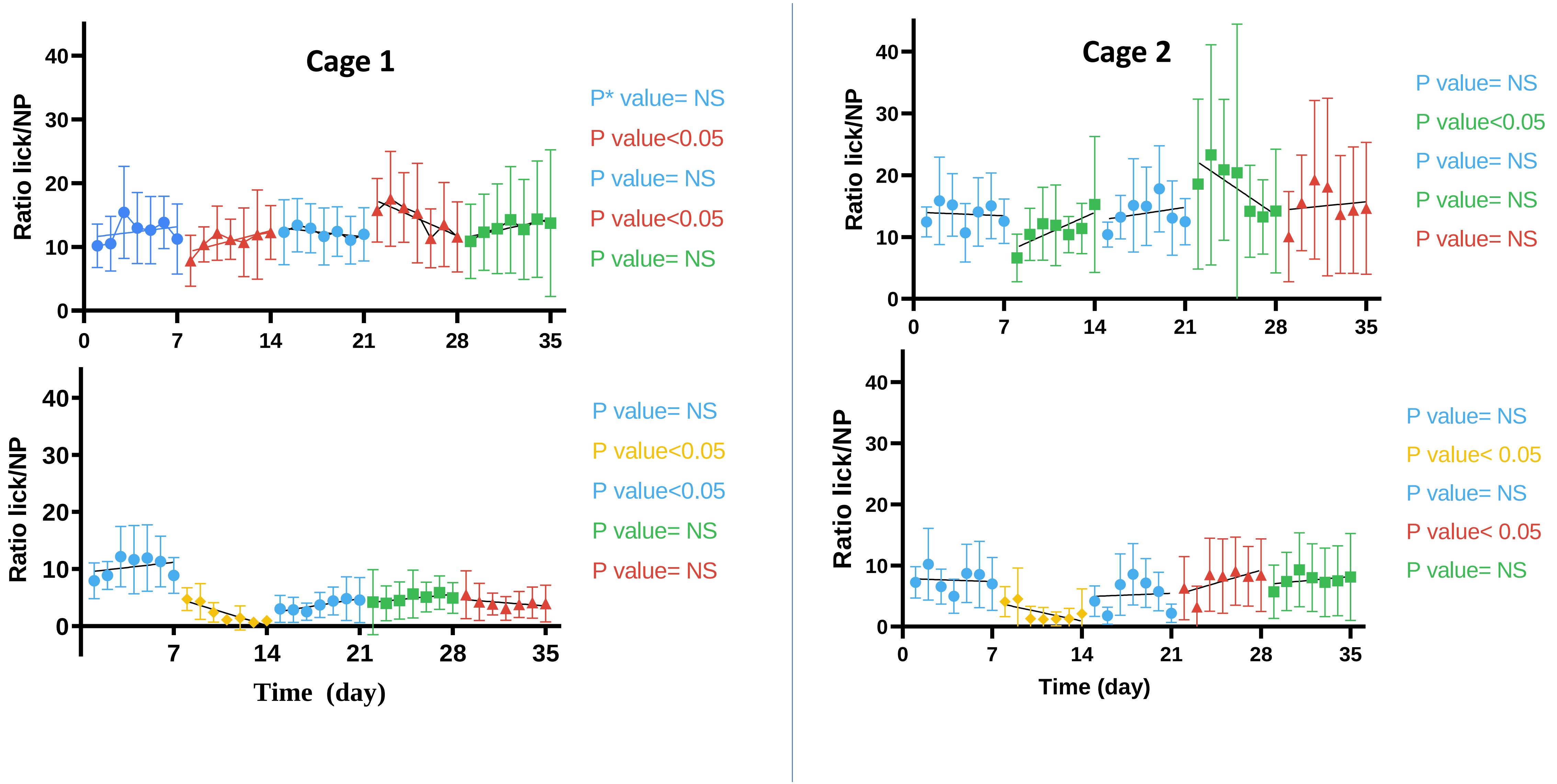

Figure S1-a and **b,** Lick/NP ratio, the number of licks normalized based on the number of NP (number of licks/ NP) in experiment 1 and 2.

* Significancy of slope from zero
